# Quarantine During COVID-19 Outbreak: Eating Behavior, Perceived Stress, and Their Independently Associated Factors in a Brazilian Sample

**DOI:** 10.3389/fnut.2021.704619

**Published:** 2021-07-26

**Authors:** Juliana Costa Liboredo, Lucilene Rezende Anastácio, Lívia Garcia Ferreira, Lívya Alves Oliveira, Ceres Mattos Della Lucia

**Affiliations:** ^1^Department of Food, Universidade Federal de Ouro Preto, Ouro Preto, Brazil; ^2^Graduate Program in Food Science, Food Science Department, Faculty of Pharmacy, Universidade Federal de Minas Gerais, Belo Horizonte, Brazil; ^3^Graduate Program in Nutrition and Health, Universidade Federal de Lavras, Lavras, Brazil; ^4^Graduate Program in Nutrition Science, Universidade Federal de Viçosa, Viçosa, Brazil

**Keywords:** feeding behavior, habits, pandemics, quarantine, life style, stress

## Abstract

The study aimed to assess the eating behavior [uncontrolled eating (UE), emotional eating (EE), and cognitive restraint (CR)], the perceived stress, and independently associated factors among Brazilians during the COVID-19 pandemic. An online survey was conducted and data about 1,368 participants were evaluated. Multivariate logistic regression models were performed to identify factors independently associated (socioeconomic, lifestyle, and eating habits data) with eating behaviors and perceived stress. Working in the COVID-19 frontline (OR = 2.19), increased food delivery (OR = 1.49), increased food intake (OR = 1.48), increased number of meals (OR = 1.13), and EE (OR = 1.05) were factors independently associated with UE. Variables that were independently associated with EE were: increased food intake (OR = 2.57), graduation in a non-health-related course (OR = 1.78), perceived stress (OR = 1.08), UE (OR = 1.07), and CR (OR = 1.02). Reduced snacking (OR = 2.08), female gender (OR = 1.47), having a higher degree (OR = 1.44), increased homemade meals (OR = 1.31), the higher difference in the frequency of instant meals and snacks intake (OR = 0.91), EE (OR = 1.01), not increased alcohol dose intake (OR = 0.57), and increased physical activity (OR = 0.54) were independently associated with CR. Perceived stress was independently associated with changes in the way of working or studying (OR = 2.48), worse sleep quality (OR = 2.22), younger age (OR = 1.06), and EE (OR = 1.02). This study indicates that socioeconomic variables, lifestyle, and eating habits were independently associated with the eating behaviors of Brazilians and perceived stress during the quarantine.

## Introduction

The acute respiratory disease caused by the SARS-CoV-2 virus (COVID-19) has already affected individuals in 220 countries, areas, or territories (1). Globally, there have been more than 175 million confirmed cases and more than 3 million deaths (1). The spread of SARS-CoV-2 led health officials worldwide to take several measures, such as complete city locking down, building hospitals, performing strict social distancing, and implementing sanitary measures. Despite the important effect against COVID-19, social distancing can lead to changes in the daily life of the population. There may be changes in access to food, in the habit of eating out (2), and even changes in food purchases by families due to the possibility of losing their jobs or having reduced income during this period (2). Food markets have access restricted and restaurants and bars have been closed, which may affect the food buying and consumption behavior (3, 4). It may further affect the choices of an individual to prepare their meals or buy premade food more often. Social distancing can affect eating patterns (5), promoting snacking, eating palatable meals, and increased alcohol consumption (6). People reported snacking more frequently (7), increased consumption of sweets and snacks rich in calories has been found in studies carried out during the quarantine in different countries (3, 5, 8).

Additionally, facing excessive daily information about the pandemic may cause stress (9). Most people usually change their eating behaviors when stressed, resulting in under or overeating, depending on the stressor severity (10). Furthermore, interruption of the work routine caused by the quarantine could result in boredom, which in turn is associated with a greater energy intake (11).

Some studies have focused on identifying eating habits change (2, 12), but information about the impact of the COVID-19 pandemic on eating behavior and perceived stress is still limited, especially in Brazil. It is believed that the pandemic has resulted in increased food delivery, food intake, number of meals, and emotional eating (EE) behavior in Brazilians. This study aimed to assess the eating behavior, the perceived stress, and their independently associated factors among Brazilians during the pandemic. These results may be useful to define strategies to minimize the problems observed and to encourage healthy behavior during the period of social distancing.

## Materials and Methods

### Study Design

**Figure 1:**
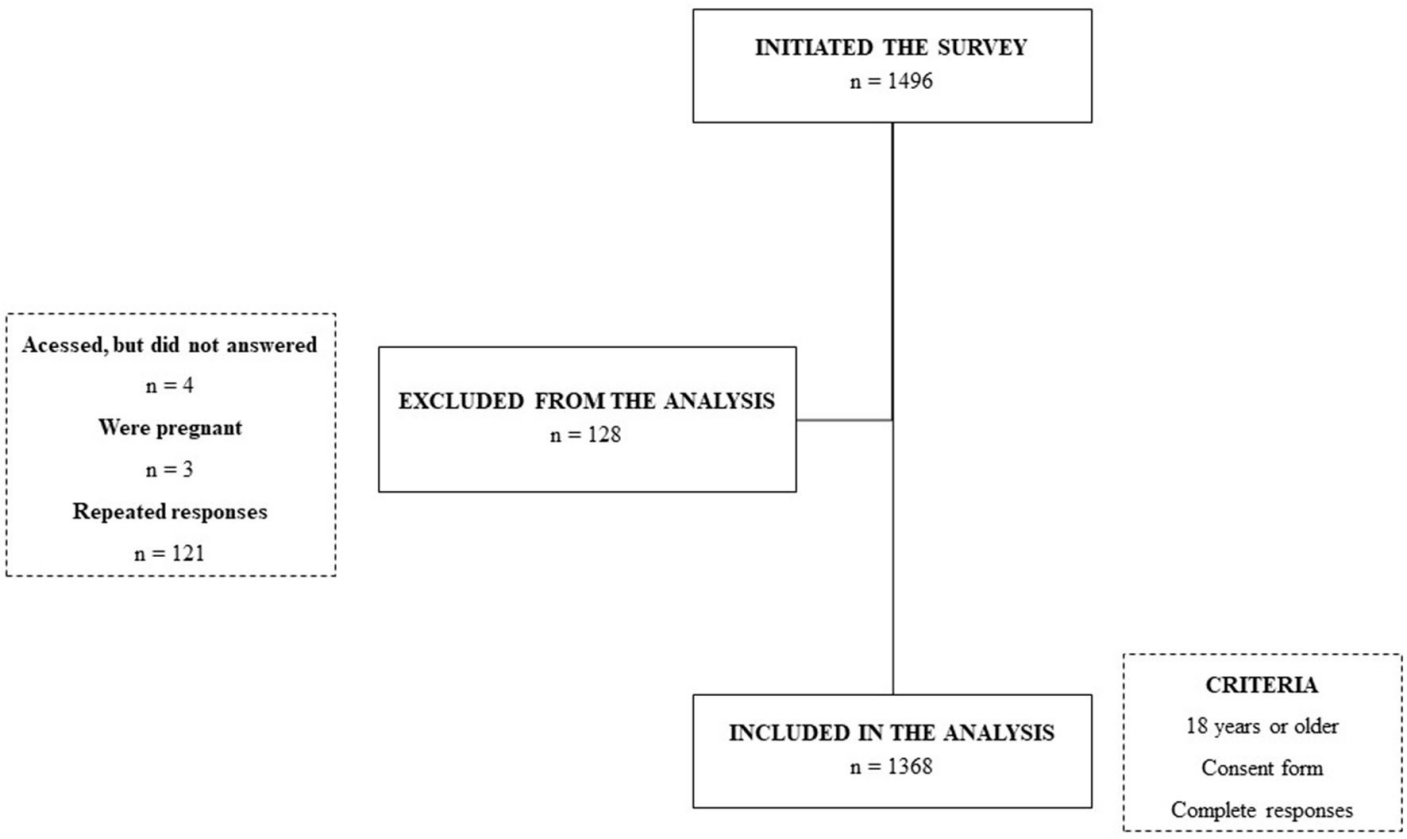
Study recruitment.

A cross-sectional online survey, based on a sample for convenience, was conducted from August 14, 2020, to September 9, 2020, approximately 5–5.5 months after the beginning of the social distancing measures in Brazil. At that time, Brazil showed the highest number of cases and deaths in Latin America, and it was the third country with the most cases in the world, behind only the United States of America and India. The lockdown measures implemented in Brazil included: suspension of non-essential activities (closing of restaurants, bars, shopping malls, and gyms), suspension of the activities of schools and universities and with the implementation of emergency remote education, the incentive to adhere to social and physical distance measures, among other issues addressed in Federal Law No. 13,.979, of February 6, 2020. Brazilian residents aged 18 years of age or older were invited to enroll in the study. Pregnant women, individuals under 18 years of age, and residents of other countries were excluded (Figure 1). The study was conducted according to the Declaration of Helsinki. The protocol was approved by the Research Ethics Committee of the Federal University of Viçosa, Minas Gerais, Brazil (Protocol number 35516720.5.0000.5153).

### Instrument and Procedure

The survey was created on Google Form Platform® and the link was shared *via* emails, university websites, and social media. The first part of the survey included the consent form. The answers of the participants were anonymous (only the emails of the participants were available), and they were able to stop their participation in the study at any stage before submitting the answers.

Eating behavior was assessed using the Brazilian version of the Three-Factor Eating Questionnaire (TFEQ-R21) translated into Portuguese and validated by Natacci and Ferreira Júnior (13). TFEQ-R21 measures eating behavior based on uncontrolled eating (UE), EE, and cognitive restraint (CR). The TFEQ consisted of 21 questions (a 4-point response format for items 1 to 20, and a numerical rating scale of 8 points for question 21). Responses to each of the questions were given a score between 1 and 4. Before calculating the scores, items 1–16 were reverse coded and item 21 was recorded as follows: 1–2 scores as 1, 3–4 as 2, 5–6 as 3, 7–8 as 4. The CR scale was composed of items 1, 5, 11, 17, 18, and 21. The UE scale was composed of items 3, 6, 8, 9, 12, 13, 15, 19, and 20. The EE scale was composed of items 2, 4, 7, 10, 14, and 16. The mean of each was calculated and transformed into a scale from 0 to 100 points as recommended in the score instruction (13). Perceived Stress (PS) was measured by a 10-item version of the Perceived Stress Scale (PSS) validated for the Brazilian population (14).

The other variables were divided into three sections: socioeconomic data, lifestyle, and eating habits. Socioeconomic data included questions about home state, gender, age, per capita income, the composition of home residents (posterior divided into groups of living with sons vs. others and living with parents vs. others), an education level (divided into groups of complete graduation course vs. incomplete graduation course or less, postgraduation or not, and graduation in a health-related course or not), current occupation (divided into professors or not, students or not, healthcare professionals or not, and workers in the line of the front of COVID-19 or not), working schedule during pandemic (perception of lower, unaltered, or greater time spent in work, including domestic activities), labor situation (alteration in the way of working or studying during a pandemic period or not, to study or work remotely full-time or part-time), and social isolation (total and partial or not).

As for lifestyle habits, participants were also asked about time and quality of sleep, amount of time of physical activity/week, smoking habit, alcohol consumption (dose and frequency), screen time (smartphones, computer, tablet, and TV) before and during the pandemic, and the differences were calculated. These variables were evaluated numerically and dichotomized into greater or not and lower or not about such habits.

Eating habits included differences in the number of meals (before and during a pandemic), the type of meals consumed (breakfast, morning snack, lunch, afternoon snack, dinner, evening snack, and other meals), amount of food, snacking (eating between meals), using food delivery service, habits of cooking at home, and food frequency (times a week) related to the periods before and during the pandemic.

A food frequency questionnaire based on the Food and Nutritional Surveillance System (SISVAN) protocol was used (15), with the following groups: legume (beans, soybeans, lentils, and chickpeas), cereal (rice, corn, and oats), bakery products (bread, cakes, and cookies), milk and dairy, fruit, meat, hamburger or canned products (hamburger, bologna, salami, and sausage), vegetables (except potatoes, cassava, and yams), sugary drinks (soft drink, canned or powdered juice, canned coconut water, guarana/blackcurrant syrup, and fruit juice with sugar), instant foods and snacks (instant noodles, packaged snacks, or crackers), candies (chocolate, pies, lollipops, gum, caramel, and jelly), and fast-food (pizza, sandwich, and finger food). The volunteers filled in information related to the time before and during pandemic for groups of foods and the differences were calculated. The frequency of consumption of the food groups was set to 0 for those who reported never consuming such food, 0.5 for those who reported consuming rarely, 1 for those who consumed once a week, 2.5 for consumption 2–3 times/week, 5 for consumption 4–6 times/week, 7 for consumption once a day, and 10 for more than once a day, and the differences between the frequency of consumption before and during the pandemic were calculated. When the differences were positive, they were classified as increased consumption and when they were negative, they were classified as decreased consumption. The consumption frequencies before and during the pandemic may be found in another study of the team (16).

Questions about lifestyle habits and eating habits were based on other online surveys performed during the COVID-19 pandemic (2, 5). To verify the adequacy and the response time of the questions, a pilot study was carried out with about 30 respondents.

### Data Analysis

Data were analyzed using the software Statistical Package for Social Sciences® (SPSS® Inc., Chicago, IL, USA) version 21.0. Data are shown as median, minimum, and maximum values for independently associated factors and interquartile intervals for UE, EE, CR, and PS. Assumption of normality was checked using the Kolmogorov-Smirnov test. The correlations between eating behaviors and PS were obtained by Spearman's correlation test. To evaluate the factors independently associated with the eating behaviors and PS, univariate (by Chi-square and Mann-Whitney) and multivariate logistic regression models, respectively, were performed. The score obtained in each questionnaire was divided into the cutoff point of the third quartile (UE: 48.1, EE: 61.1, CR: 61.1, and PS: 28.0). The highest quartile was chosen because it represents 25% of the most extreme data. The same was performed by other authors that used some scales related to eating behavior (17, 18). The models were obtained by the backward method. The fit of the models was tested by the Hosmer-Lemeshow test (*p* > 0.05). The level of significance adopted was 0.05.

## Results

A total of 1,368 individuals were enrolled in this study (1,496 answers were computed, but four individuals submitted the questionnaire and did not answer, three women were pregnant and there were 121 repeated answers). The responders were from the five regions of Brazil, but most participants (89.6%) reside in the southeast region.

### Socioeconomic, Lifestyle, and Eating Habits Status

The median age of volunteers was 31 years old (varying from 18–87), and most responders were female (80%). Regarding the profile of home residents, 38.3% lived with parents and 25.1% lived with children. Most of the responders were graduates (65.9%), 46.5% attended postgraduation courses, and 49.6% were graduates in a health-related course. Most participants of the study reported increased time spent at work (65.9%), to be working or studying remotely full or partial-time (70.7%), and changes in the way of working or studying during the pandemic (89%). Furthermore, 57.2% related total social isolation during the pandemic.

Many respondents reported decreased sleep time (31.1%) and worsened sleep quality (46.3%) during quarantine. There was a positive difference in screen time with a median of 3.5 h, and 64.6% of participants showed longer screen time during the pandemic. The frequency of alcoholic beverages intake was increased by 17.9% of individuals, but 18.6% reported reduced frequency consumption and 20.3% increased the dose of alcoholic beverages consumed. Only 1.2% of the respondents related to increasing cigarette use and 43.3% reduced the physical activity time.

Regarding eating habits, food intake increased in 58.6% of the participants, and 51.5% reported snacking more frequently. The use of delivery food and homemade meals increased by 50.1 and 67.3%, respectively (Table 1).

**Table 1:** Socioeconomic factors, lifestyle, and eating habits of a Brazilian sample during the COVID-19 pandemic period.

**Variables**	**% (*n*)^a^** **Median (min-max)^b^**
Gender	
Female	80.0 (1,094)
Male	19.7 (269)
Age (years)	31.0 (18.0–87.0)
Per capita income ($)^c^	334.6 (15.9–3059.0)
Home residents	
Living with children	25.1 (344)
Living with parents	38.3 (524)
Education level	
Graduate or above	65.9 (902)
Undergraduate or below	33.9 (464)
Post-graduation	46.5 (636)
Health-related graduation course	49.6 (679)
Profession	
Student	45.3 (620)
Healthcare worker	19.4 (265)
Professor	17.3 (237)
COVID-19 frontline worker	6.1 (83)
Time spent at work (including household chores)	
Reduced	12.8 (175)
The same	21.3 (291)
Increased	65.9 (902)
Labor situation	
Full-time work/study	89.0 (1218)
Full/part-time work or study	40.6 (555)
Changes in the way of working or studying	70.7 (967)
Social isolation	
Total	57.2 (783)
Partial	39.8 (544)
No	3.0 (41)
Sleep time difference (hours)	0.0 (−5.5 to 8.0)
Increased sleep hours	43.1 (590)
Reduced sleep hours	31.1 (425)
Improved sleep quality	13.3 (182)
Worsened sleep quality	46.3 (634)
Screen time difference (hours)	3.5 (−8.0 to 14.0)
Increased screen time	64.6 (883)
Reduced screen time	2.0 (28)
Alcoholic beverage difference (times/week)	0.0 (−7.0 to 7.0)
Increased frequency of alcoholic beverage intake	17.9 (245)
Reduced frequency of alcoholic beverage intake	18.6 (254)
Difference in dose of alcoholic beverage intake	0.0 (−6.0 to 6.0)
Increased dose of alcoholic beverage intake	20.3 (275)
Reduced dose alcoholic beverage intake	11.7 (159)
Difference in number of cigarettes	0.0 (−10.0 to 32.0)
Increased number of cigarettes	1.2 (16)
Reduced number of cigarettes	0.5 (7)
Difference in physical activity (min)	0.0 (−280.0 to 280.0)
Increased physical activity	20.8 (285)
Reduced physical activity	43.3 (593)
Use of medication	40.4 (552)
Difference in number of meals	0.0 (−6.0 to 5.0)
Increased number of meals	23.1 (316)
Reduced number of meals	26.6 (364)
Increased food intake	58.6 (802)
Reduced food intake	16.2 (221)
Increased snacking	51.5 (704)
Reduced snacking	8.0 (110)
Increased using food delivery service	50.1 (686)
Reduced using food delivery service	13.5 (185)
Increased homemade meals	67.3 (921)
Reduced homemade meals	5.8 (80)

a *Chi-square was used in univariate analyses in category variables*.

b *Mann-Whitney was used in univariate analyses in continuous variables*.

c *R$1 = $0.18 (current value)*.

### Eating Behaviors and Perceptive Stress: Independently Associated Factors

The respective median scores for UE, EE, and CR were 33.9 (interquartile interval: 18.5–48.1), 44.5 (interquartile interval: 27.8–61.1), and 39.5 (interquartile interval: 11.1–61.1) points. PS showed a median of 22.9 (interquartile interval: 19.0–28.0) points. The UE behavior was significantly and positively correlated to all the other behaviors and PS (EE: *r* = 0.098; CR: r = 0.714; PS: *r* = 0.257; *p* < 0.001 for all) and so the EE behavior (CR: *r* = 0.171 and PS: *r* = 0.334). However, the correlation between CR behavior and PS was significantly negative (*r* = −0.030). Variables associated with UE, EE, CR, and PS above the third quartile by univariate analyses are found in the Supplementary Material.

**Figure 2:**
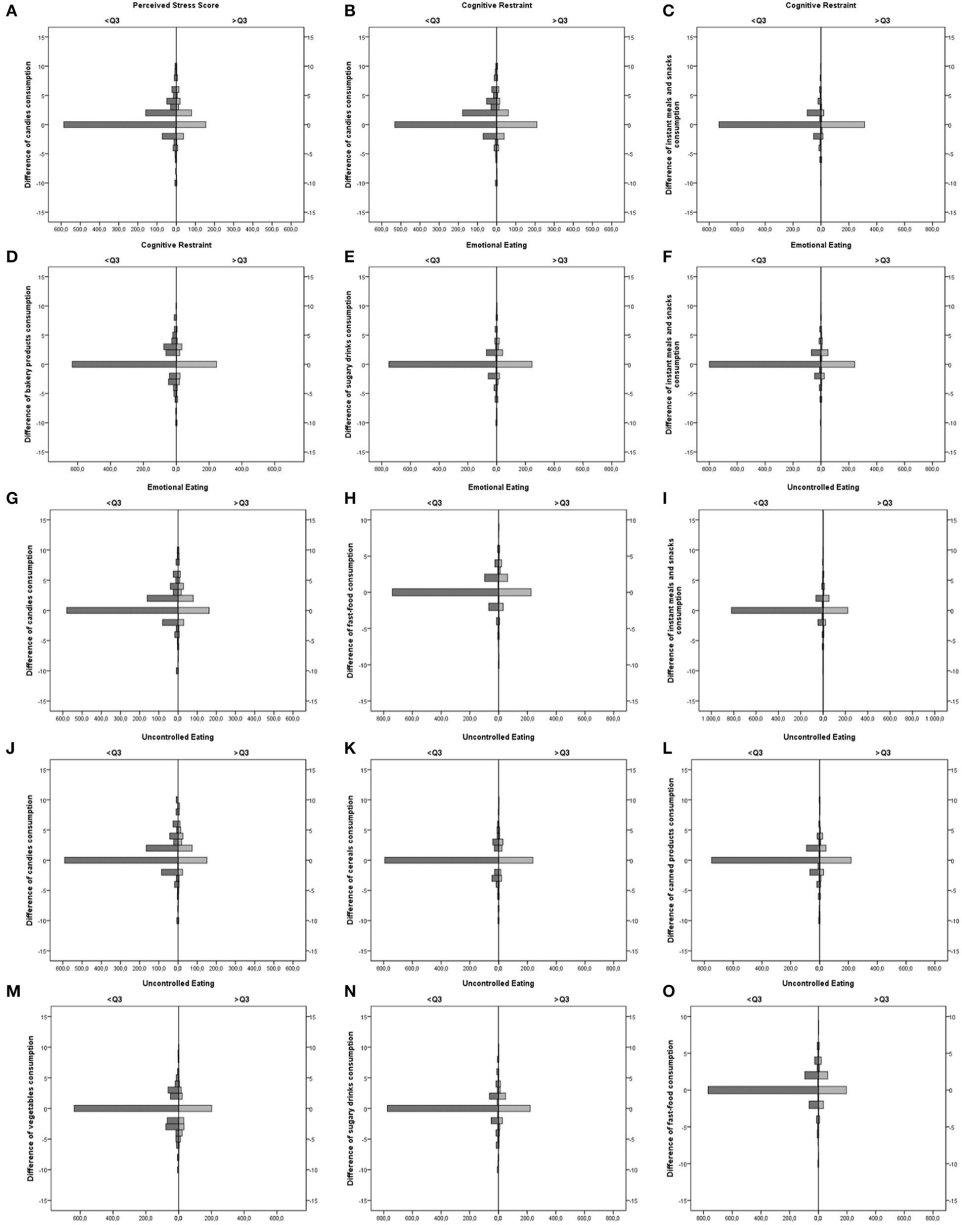
Difference of food consumption among Brazilians who were under and above the third quartile of perceived stress, cognitive restraint (CR), emotional eating (EE), and uncontrolled eating (UE) scores. **(A)** Difference of candies consumption among Brazilian who were below and above the 3rd Quartile of Perceived Stress score; **(B)** Difference of candies consumption among Brazilian who were below and above the 3rd Quartile of Cognitive Restraint score; **(C)** Difference of instant meals and snacks consumption among Brazilian who were below and above the 3rd Quartile of Cognitive Restraint score; **(D)** Difference of bakery products consumption among Brazilian who were below and above the 3rd Quartile of Cognitive Restraint score; **(E)** Difference of sugary drinks consumption among Brazilian who were below and above the 3rd Quartile of Emotional Eating score; **(F)** Difference of instant meals and snacks consumption among Brazilian who were below and above the 3rd Quartile of Emotional Eating score; **(G)** Difference of candies consumption among Brazilian who were below and above the 3rd Quartile of Emotional Eating score; **(H)** Difference of fast-food consumption among Brazilian who were below and above the 3rd Quartile of Emotional Eating score; **(I)** Difference of instant meals and snacks consumption among Brazilian who were below and above the 3rd Quartile of Uncontrolled Eating score; **(J)** Difference of candies consumption among Brazilian who were below and above the 3rd Quartile of Uncontrolled Eating score; **(K)** Difference of cereals consumption among Brazilian who were below and above the 3rd Quartile of Uncontrolled Eating score; **(L)** Difference of canned products consumption among Brazilian who were below and above the 3rd Quartile of Uncontrolled Eating score; **(M)** Difference of vegetables consumption among Brazilian who were below and above the 3rd Quartile of Uncontrolled Eating score; **(N)** Difference of sugary drinks consumption among Brazilian who were below and above the 3rd Quartile of Uncontrolled Eating score; **(O)** Difference of fast-food consumption among Brazilian who were below and above the 3rd Quartile of Uncontrolled Eating score.

The significant difference in the frequency of food consumption (before and during the pandemic period) among the respondents who were under and above the third quartile of PS, CR, EE, and UE scores are shown in Figure 2.

The multiple logistic regression analysis is summarized in Table 2. Factors that independently increased the probability of being in the highest quartile of UE score were: being a COVID-19 frontline worker (OR: 2.196; CI: 1.233–3.911), increased food delivery (OR: 1.498; CI: 1.105–2.031), increased food intake (OR: 1.483; CI: 1.049–2.097), increased number of meals (OR: 1.483; CI: 1.049–2.097), and emotional eating (OR: 1.054; CI: 1.047–1.061). Increased food intake (OR: 2.579; CI: 1.817–3.660), graduation in another non-health-related course (OR: 1.785; CI: 1.305–2.443), PS (OR: 1.080; CI: 1.052–1.108), UE (OR: 1.078; CI: 1.068–1.088), and CR (OR: 1.020; CI: 1.012–1.028) increased the probability of being in the highest quartile of EE score. Finally, factors positively and independently associated with CR were: reduced frequency of snacking (OR: 2.080; CI: 1.376–3.144), female gender (OR: 1.468; CI: 1.447–2.053), graduate status (OR: 1.443; CI: 1.097–1.893), increased frequency of homemade meals (OR: 1.314; CI: 1.004–1.722), increased difference in the frequency of ultra-processed food intake (OR: 0.91; CI: 0.849–0.981), and EE score (OR: 1.007; CI: 1.003–1.012). On the other hand, unincreased alcohol dose intake (OR: 0.573; CI: 0.405–0.811) and increased physical activity time (OR: 0.537; CI: 0.402–0.711) decreased the chance of being in the highest quartile of CR score. About PS, factors independently associated were: changes in the way of working or studying during quarantine (OR: 2.480; CI: 1.470–4.183), worsened sleep quality (OR: 2.222; CI: 1.700–2.904), younger age (OR: 1.069; CI: 1.053–1.085), and EE (OR: 1.016; CI: 1.012–1.021).

**Table 2:** Factors independently associated with the last quartile of the eating behaviors and stress among Brazilians during the pandemic period in multivariate analyses.

**Behaviors**	**OR**	**CI (95%)**		***P*-value**
		**Lower**	**Upper**	
**Uncontrolled eating (82.0% of prediction; Hosmer Lemeshow test** **=** **0.278)**				
Frontline worker	2.196	1.233	3.911	0.008
Increased using food delivery service	1.498	1.105	2.031	0.009
Increased food intake	1.483	1.049	2.097	0.026
Increased number of meals	1.135	1.003	1.284	0.044
Emotional eating	1.054	1.047	1.061	<0.001
Constant	0.016			<0.001
**Emotional eating (83.2% of prediction; Hosmer Lemeshow test** **=** **0.356)**				
Increased food intake	2.579	1.817	3.660	<0.001
Graduation in a non-health-related course	1.785	1.305	2.443	<0.001
Perceived stress	1.080	1.052	1.108	<0.001
Uncontrolled eating	1.078	1.068	1.088	<0.001
Cognitive restraint	1.020	1.012	1.028	<0.001
Constant	0.001			<0.001
**Cognitive restraint (72.7% of prediction; Hosmer Lemeshow test** **=** **0.099)**				
Reduced snacking	2.080	1.376	3.144	0.001
Female gender	1.468	1.447	2.053	0.024
Graduate or above	1.443	1.097	1.893	0.009
Increased homemade meals	1.314	1.004	1.722	0.047
Higher difference in ultraprocessed food	0.916	0.849	0.981	0.017
Emotional eating	1.007	1.003	1.012	0.001
No increase in alcohol dose intake	0.573	0.405	0.811	0.002
Increased physical activity (hours practiced)	0.537	0.402	0.711	<0.001
Constant	0.323			<0.001
**Perceived stress (75.4% of prediction; Hosmer Lemeshow test** **=** **0.851)**				
Change in the way of work or study during pandemic period	2.480	1.470	4.183	0.001
Worsened sleep quality	2.222	1.700	2.904	<0.001
Younger age	1.069	1.053	1.085	<0.001
Emotional eating	1.016	1.012	1.021	<0.001
Constant	0.412			0.020

## Discussion

The findings revealed that many respondents showed altered habits during the COVID-19 pandemic. Sleeping changes and physical activity reduction were observed, as found by Ammar et al. (12). Increased number of daily meals, increased food intake, snacking, increased use of food delivery, and homemade meals were also verified. Likewise, changes in diet during the pandemic have also been previously reported (2, 5). Studies carried out with Brazilian samples also found negative changes in eating habits (19, 20).

Furthermore, people during quarantine might experience severe disturbances in eating behavior, such as extremely reduced food intake or overeating, which could increase body weight and shape concerns (21). The lack of data about the eating behavior of Brazilians during the COVID-19 pandemic and the use of different questionnaires in other populations during quarantine limit comparisons. However, volunteers of the present research reported similar scores of EE [44.5 (27.8–61.1)] and lower scores of UE [33.9 (18.5–48.1)] and CR [39.5 (11.1–61.1)] in comparison to the study of de Medeiros et al. (22), performed before the pandemic, with healthy adult Brazilians (EE: 45.8 ± 29.2; UE: 55.6 ± 25.0, and CR: 70.8 ± 25.0). However, Papandreou et al. (23) found higher scores of eating behaviors in the Spanish and Greek population than other pre-COVID-19 data, supporting the idea that these behaviors may be affected during quarantine. Elmacioglu et al. (24), in a study that evaluated the eating behavior during the social isolation in the COVID-19 pandemic, found an increase in the EE and UE of individuals, but no significant changes in CR occurred.

In this study, the number of meals, food intake, and use of food delivery increased the chance to have UE scores in the highest quartile in 13.5, 48.3, and 49.8%, respectively. Increased food consumption and changes in eating behaviors are frequent in subjects ordered to follow stay-at-home during the pandemic (5). In the present study, the highest quartile of the behaviors studied was associated with differences in the frequency of candies (PS, CR, UE, and EE), instant meals and snacks (CR, UE), bakery products (CR), sugary drinks (EE, UE), cereals (UE), canned product (UE), vegetables (UE), and fast-food (EE, UE) consumption (Figure 2).

Furthermore, COVID-19 frontline workers showed a 2.2 higher chance to have greater UE scores. Beyond the risk of exposure to COVID-19 by contact with patients and coworkers, healthcare workers are under increasing stress and mental health risks due to higher workload, shortages of protective equipment (25), death of their colleagues after exposure to the virus, and fear of spreading it to their families (26). The context experienced by these workers may probably have contributed to the higher UE scores observed in this study.

Individuals with higher scores of EE showed higher chances to have UE scores above the third quartile. Studies have attempted to identify the influence of emotions on food consumption (27, 28). Humor and emotions can influence food choice, in the same way, that consumption of certain foods can change a mood or emotional state (27). People who have EE seem to be more susceptible to the effects of stress and may try to obtain comfort through food (29). Stress may promote irregular eating patterns and strengthen networks toward hedonic overeating, choosing more pleasurable, and palatable foods irrespective of caloric intake changes (30). In an Italian study (9) carried out during the COVID-19 pandemic, almost half of respondents felt anxious about their eating habits, consumed comfort foods, and increased food intake to feel better.

Emotional eating is related to the tendency of individuals to overconsumption of food in response to negative emotional stimuli (13). Increased food intake and UE, CR, and PS were associated with higher scores of EE. These results indicate that EE is determined by other eating behaviors and by PS, and probably affected food choice, as the strong association among EE and an increase in food intake during quarantine. Emotional factors probably impede the control of food intake in situations of stress. The EE behavior can occur as a coping strategy concerning negative emotions (31). Other authors reported the association between stress and eating behavior of EE (30, 31). In a study carried out during the COVID-19 outbreak, in which the majority (73.6%) of the participants reported moderate to high levels of perceived stress, the EE was significantly correlated with four of the nine reasons for food choice: mood, convenience, price, and familiarity (32). In a study conducted with mothers from Los Angeles, California, the authors observed that the most common strategy that mothers indicated to deal with stress related to COVID-19 was to eat comfort foods (e.g., sweets and snacks) (58.7%). The PS related to COVID-19 was positively associated with the BMI of the mother and emotional eating (33).

In the present study, EE was a factor independently associated with CR, increasing the chance of individuals to show a higher CR score. A positive correlation between CR and EE was also previously observed in a cohort study (34), reporting that the CR impairment can leave the individual vulnerable to emotional eating and more reactive to sensory or cognitive exposure linked to food. The cognitively restricted individual imposes a set of dietary obligations and prohibitions to maintain or lose weight, but many of them, when exposed to certain situations, such as stress, tend to overeat (28). Restrained eaters may become hypervigilant to threat stimuli, accentuated by having to remember these during eating, at the expense of maintaining self-awareness or monitoring dietary concerns and goals (35).

In this study, individuals who practiced more physical activity and those who did not increase their alcohol dose intake showed 46.3 and 42.7% less probability of being CR scores above the third quartile, respectively. CR has been previously associated with higher cortisol levels which can activate the stress response (36). It is well-recognized that sustained exercise may influence basal cortisol levels (37) and might affect the response to stressors (38). Although, high restraint scores have been associated with more hours of weekly exercise (39), these findings have not been shown in the study. The relation between alcohol intake and CR has been reported previously, as restrained behaviors of individuals show disinhibition/impulsive episodes of food eating as the binge drinking ones (40). Hunt and Forbush (41) found that CR predicted the presence of alcohol misuse and drunkorexia in college students.

In contrast to what was expected from the pandemic, increased food intake was not associated with CR. Other factors such as female gender, graduation, reduced snacking, and increased frequency of homemade meals to the period before the pandemic increased the probability of individuals having higher CR scores. On the other hand, a higher frequency of ultra-processed food intake reduced the probability of higher CR scores. Regarding gender, studies have shown that many young women are motivated to both obtain and maintain their perceived ideal body shape (42). Furthermore, women who constantly monitor their bodies tend to put an extreme emphasis on outward appearance and weight and are also more likely to be motivated in adopting stressful behaviors to obtain a body that meets societal expectations (43).

In relation to PS, the multivariate model indicated that changes in the way of working or studying in relation to the period prior to the pandemic showed a 2.48 higher chance to produce PS scores above the third quartile. The results showed that most participants related changes in their way of work or study and increased time spent at work. During the pandemic, corporations and governments encouraged the practice of working from home to reduce exposure to COVID-19 (44). However, the responsibilities of people were amplified, including teleworking, doing domestic activities, minding their children, and facilitating homeschooling (44).

Sleep quality, younger age, and higher EE score were also factors independently associated with PS. Sleep deprivation is a common chronic stressor that may contribute to an increased risk of obesity and metabolic diseases (45). However, long-term studies that may assess the relationship between stress during the pandemic and the outcomes of chronic disorders have not yet been published. Additionally, the social isolation necessary to flatten the epidemic curve restricts young people from having physical contact with friends, causing increased feelings of loneliness, and stress (46). The relationship between stress and emotional eating has been discussed previously in this study.

PS showed a median of 22.9 (interquartile interval: 19.0–28.0) points. During the pandemic, other studies showed moderate (total mean scores between 14 and 26) or high- stress levels (total mean scores between 27 and 40) in most participants (32, 47). Considering these cutoff values, 88% of the volunteers showed moderate or high-stress levels, and 26.1%, high levels. COVID-19 is a stressor with great impact and unknown long-term implications, but may not be stressful or cause the same degree of stress for everyone (48).

The main limitation of this study is related to the lack of data about the eating behavior of participants and PS before the pandemic or the lack of a comparison group that has not been through isolation during the COVID-19 outbreak. Longitudinal studies are necessary to explain better the association of the pandemic between eating behavior and perceived stress. The studied sample may not be representative of the entire Brazilian population since the number of respondents was higher in the Southeast region and most participants were women. Also, other limitations may involve the self-reported data and the fact that only individuals who had access to a computer and were technically savvy may have taken this study. Despite these limitations, this research contributes to a better understanding of pandemic effects on eating behavior and stress.

We observed that many individuals experienced modification of their habits, manifested by reduced sleeping time, worsened sleep quality, increased frequency in consuming alcoholic beverages, reduced physical activity, and increased food intake. Working in the pandemic and increased food delivery were some of the factors associated with the eating behaviors. Perceived stress was associated with changes in the way of working or studying and by worse sleep quality.

## Data Availability Statement

The datasets presented in this article are not readily available because the database is being used for other studies by the research group that has not yet been published. Requests to access the datasets should be directed to Ceres Mattos Della Lucia, cmdellalucia@ufv.com.

## Ethics Statement

The studies involving human participants were reviewed and approved by Research Ethics Committee of the Federal University of Viçosa, Minas Gerais, Brazil (Protocol number 35516720.5.0000.5153). The patients/participants provided their written informed consent to participate in this study.

## Author Contributions

JL, LA, LF, LO, and CD participated in the design of the project, analysis, and interpretation of the data. The orientation and critical review of the content were carried out by JL, LA, LF, and CD. All the authors were responsible for the final approval of the version to be published.

## Conflict of Interest

The authors declare that the research was conducted in the absence of any commercial or financial relationships that could be construed as a potential conflict of interest.

## Publisher's Note

All claims expressed in this article are solely those of the authors and do not necessarily represent those of their affiliated organizations, or those of the publisher, the editors and the reviewers. Any product that may be evaluated in this article, or claim that may be made by its manufacturer, is not guaranteed or endorsed by the publisher.
