# Supplementary material for: Quarantine During COVID-19 Outbreak: Eating Behavior, Perceived Stress, and Their Independently Associated Factors in a Brazilian Sample
Source: Front Nutr. 2021 Jul 26;8:704619. doi: 10.3389/fnut.2021.704619 (PMC8349978; doi:10.3389/fnut.2021.704619)
Supplement: Supplementary file 1 [file Data_Sheet_1.docx]

Supplementary Material

**Table 1.** Socioeconomic factors associated with eating behavior (uncontrolled eating, emotional eating and cognitive restriction) and stress among Brazilians during pandemic period in univariate analyses

| **Variables** | **Eating behavior** | | | | | | | **Perceived**  **Stress** | |  |
| --- | --- | --- | --- | --- | --- | --- | --- | --- | --- | --- |
|  | **Uncontrolled eating** | | | **Emotional eating** | | **Cognitive restraint** | |  | |  |
|  | **< 48.1** | **≥48.1** | **<61.1** | | **≥61.1** | **<61.1** | **≥61.1** | **<28.0** | **≥28.0** | |
| **Gender** |  |  |  | |  |  |  |  |  | |
| Female | 78.7 (789) | 84.7 (305)^*^ | 77.2 (757) | | 88.2 (337)^**^ | 78.3 (763) | 85.1 (331)^*^ | 78.2 (787) | 86.2 (307)^*^ | |
| Male | 21.3 (214) | 15.3 (55) | 22.8 (224) | | 11.8 (45) | 21.7 (211) | 14.9 (58) | 21.8 (220) | 13.8 (49) | |
| **Age (years)** | 31.0  (18.0-87.0) | 30.5  (18.0-66.0) | 32.0  (18.0-87.0) | | 30.0  (18.0-66.0)^*^ | 31.0  (18.0-87.0) | 32.0  (18.0-68.0)^*^ | 33.0  (18.0-87.0) | 26.0  (18.0-62.0)^*^ | |
| **Per capita income ($)^3^** | 334.6  (15.9-1229.4) | 334.6  (19.2-1529.5) | 358.5  (15.9-3059.0) | | 286.8^*^  (15.9-2389.9) | 334.6  (15.9-3059.0) | 358.5  (15.9-2389.9) | 358.5  (87.1-3059.0) | 234.0  (15.9-2389.9) | |
| **Home residents** | |  |  | |  |  |  |  |  | |
| Living with children | 24.9 (251) | 25.8 (93) | 24.7 (243) | | 26.4 (101) | 23.8 (233) | 28.5 (111) | 28.5 (288) | 15.7 (56) | |
| Living with parentes | 37.9 (382) | 39.4 (142) | 37.4 (368) | | 40.7 (156) | 60.8 (595) | 64.0 (249) | 33.8 (342) | 51.0 (182)^**^ | |
| **Education level** | |  |  | |  |  |  |  |  | |
| Graduate or above | 66.2 (666) | 65.6 (236) | 66.9 (658) | | 63.9 (244) | 63.7 (622) | 72.0 (280)^*^ | 70.6 (712) | 53.2 (190) | |
| Undergraduate or below | 33.8 (340) | 34.4 (124) | 33.1 (226) | | 36.1 (138) | 36.3 (355) | 28.0 (109) | 29.4 (297) | 46.8 (167)^**^ | |
| Post-graduation | 45.8 (462) | 48.3 (174) | 53.8 (530) | | 52.7 (202) | 45.4 (444) | 49.4 (192) | 49.4 (499) | 38.4 (137) | |
| Health-related graduation course | 50.8 (512) | 46.4 (167) | 52.3 (515) | | 42.8 (164) | 49.5 (485) | 49.9 (194) | 48.3 (488) | 53.5 (191) | |
| **Profession** |  |  |  | |  |  |  |  |  | |
| Student | 45.1 (455) | 45.8 (165) | 45.1 (444) | | 46.0 (176) | 46.3 (453) | 42.9 (167) | 39.8 (402) | 61.1 (218)^**^ | |
| Healthcare worker | 20.0 (202) | 17.5 (63) | 20.1 (198) | | 17.5 (67) | 19.0 (186) | 20.3 (79) | 21.1 (213) | 14.6 (52) | |
| Professor | 17.1 (172) | 18.1 (65) | 16.9 (166) | | 18.5 (71) | 16.3 (160) | 19.8 (77) | 18.4 (186) | 14.3 (51) | |
| COVID-19 frontline worker | 25.4% (327) | 39.8% (33)^**^ | 68.7% (57) | | 31.3% (26) | 67.5% (56) | 32.5% (27) | 83.1% (69) | 16.9% (14) | |
| **Time spent at work (including household chores)** | | | | |  |  |  |  |  |  |
| Reduced | 13.0 (131) | 12.2 (44) | 12.7 (125) | | 13.1 (50) | 13.0 (127) | 12.3 (48) | 12.0 (121) | 15.1 (54) | |
| The same | 22.6 (228) | 17.5 (63) | 22.5 (222) | | 18.0 (69) | 22.0 (215) | 19.5 (76) | 24.3 (246) | 12.6 (45) | |
| Increased | 64.4 (649) | 70.3 (253)^*^ | 64.8 (638) | | 68.9 (264) | 65.1 (637) | 68.1 (265) | 63.7 (644) | 72.3 (258)^**^ | |
| **Labor situation** | |  |  | |  |  |  |  |  | |
| Full-time work study remotely | 90.1 (908) | 86.1 (310) | 90.3 (889) | | 85.9 (329) | 89.9 (880) | 86.9 (338) | 87.0 (880) | 94.7 (338)^**^ | |
| Full/part-time work or study remotely | 40.0 (403) | 42.2 (152) | 41.1 (405) | | 39.2 (150) | 41.5 (406) | 38.3 (149) | 38.9 (393) | 45.4 (162)^*^ | |
| Changes in the way of working or studying | 71.0 (716) | 69.7 (251) | 71.4 (703) | | 68.9 (264) | 72.0 (705) | 67.4 (262) | 68.2 (689) | 77.9 (278)^*^ | |
| **Social isolation** | |  |  | |  |  |  |  |  | |
| Total | 58.8 (593) | 52.8 (190) | 59.3 (584) | | 52.0 (199) | 58.4 (572) | 54.2 (211) | 55.0 (556) | 63.6 (227)^*^ | |
| Partial | 38.6 (389) | 43.1 (155) | 37.9 (373) | | 44.6 (171)^*^ | 38.2 (374) | 43.7 (170) | 41.5 (420) | 34.7 (124) | |
| No | 2.6 (26) | 4.2 (15) | 2.8 (28) | | 3.4 (13) | 3.4 (33) | 2.1 (8) | 3.5 (35) | 1.7 (6) | |

^1^ Mann-whitney was used in univariate analyses in continuous variables ^2^ Qui-square was used in univariate analyses in category variables ^3^ $1 = R$0.18 (current value)

**Table 2.** Lifestyle habits factors associated to eating behavior (uncontrolled eating, emotional eating and cognitive restrain) and stress among Brazilians during pandemic period in univariate analyses

| **Variables** | **Eating behavior** | | | | | | **Perceived**  **Stress** | |
| --- | --- | --- | --- | --- | --- | --- | --- | --- |
|  | **Uncontrolled eating** | | **Emotional eating** | | **Cognitive restraint** | |  | |
|  | **< 48.1** | **≥48.1** | **<61.1** | **≥61.1** | **<61.1** | **≥61.1** | **<28.0** | **≥28.0** |
| **Sleep time difference (hours)** | 0.00  (-5,5.0 - 8.0) | 0.00  (-5.0 - 4.50) | 0.00  (-5.5 – 8.0) | 0.00^*^  (-5.0 – 7.0) | 0.00  (-5.0 – 8.0) | 0.00  (-5.5 – 7.3) | 0.00  (-5.0 – 8.0) | 0.0  (-5.5 – 5.5) |
| **Increased sleep hours** | 44.0 (440) | 42.7 (150) | 44.6 (435) | 41.2 (155) | 43.8 (424) | 43.1 (166) | 44.0 (440) | 42.6 (150) |
| **Reduced sleep hours** | 29.9 (301) | 34.4 (124) | 28.9 (285) | 36.6 (140)^*^ | 32.2 (315) | 28.3 (110) | 28.5 (288) | 38.4 (137)^*^ |
| **Improved sleep quality** | 12.9 (130) | 14.5 (52) | 14.3 (141) | 10.7 (41) | 12.7 (124) | 14.9 (58) | 13.5 (136) | 12.9 (46) |
| **Worsened sleep quality** | 43.8 (441) | 53.8 (193)^*^ | 42.0 (413) | 57.7 (221)^**^ | 46.2 (452) | 48.8 (182) | 40.3 (407) | 63.6 (227)^**^ |
| **Screen time difference (hours)** | 3.5  (-7.5 – 14.0) | 3.5  (-8.0 – 14.0) | 3.5  (-7.5 – 14.0) | 3.5  (-8.0 – 14.0) | 3.5  (-4.0 – 14.0) | 3.5  (-8.0 – 14.0) | 3.5  (-7.5 – 14.0) | 4.0  (-8.0 – 14.0) |
| **Increased screen time** | 63.8 (643) | 66.7 (240) | 64.5 (635) | 64.8 (248) | 63.6 (623) | 66.8 (260) | 63.4 (641) | 67.8 (242) |
| **Reduced screen time** | 2.2 (22) | 1.7 (6) | 2.6 (26) | 0.5 (2) | 2.3 (23) | 1.3 (5) | 1.9 (19) | 2.5 (9) |
| **Alcoholic beverage difference (times/week)** | 0.00  (-6.5 – 7.0) | 0.00  (-7.0 – 6.0) | 0.00  (-6.5 – 7.0) | 0.00  (-7.0 – 4.5) | 0.00  (-7.0 – 7.0) | 0.000  (-6.5 – 6.0) | 0.000  (-7.0 – 7.0) | 0.000  (-6.5 – 4.5) |
| **Increased frequency of alcoholic beverage intake** | 16.0 (161) | 23.3 (84)^*^ | 15.5 (153) | 24.0 (92)^**^ | 17.2 (168) | 19.8 (77) | 17.3 (175) | 19.6 (70) |
| **Reduced frequency of alcoholic beverage intake** | 18.1 (182) | 20.0 (72) | 18.1 (178) | 19.8 (76) | 20.5 (201) | 13.6 (53) | 16.0 (162) | 25.8 (92)^**^ |
| **Difference in dose of alcoholic beverage intake** | 0.00  (-6.0 – 6.0) | 0.00  (-6.0 – 4.0) | 0.00  (-6.0 – 6.0) | 0.00  (-6.0 – 4.0) | 0.00  (-6.0 – 6.0) | 0.00  (-6.0 – 4.0) | 0.00  (-6.0 – 6.0) | 0.00  (-6.0 – 4.0) |
| **Increased dose of alcoholic beverage intake** | 19.9 (199) | 21.5 (76) | 19.0 (186) | 23.5 (89) | 22.1 (215) | 15.6 (60) | 18.0 (180) | 26.9 (95)^*^ |
| **Reduced dose alcoholic beverage intake** | 11.0 (110) | 13.9 (49) | 11.1 (108) | 13.5 (51) | 11.3 (110) | 12.8 (49) | 11.4 (114) | 12.7 (45) |
| **Difference in number of cigarettes** | 0.00  (-10.0 – 32.0) | 0.00  (-10.0 – 25.0) | 0.00  (-10.0 – 32.0) | 0.00  (-10.0 – 25.0) | 0.00  (-10.0 – 32.0) | 0.00  (-5.0 – 15.0) | 0.00  (-10.0 – 32.0) | 0.00  (-10.0 – 5.0) |
| **Increased number of cigarettes** | 1.1 (11) | 1.4 (5) | 1.2 (12) | 1.0 (4) | 1.2 (12) | 1.0 (4) | 1.4 (14) | 0.6 (2) |
| **Reduced number of cigarettes** | 0.4 (4) | 0.8 (3) | 0.5 (5) | 0.5 (2) | 0.6 (6) | 0.3 (1) | 0.2 (2) | 1.4 (5)^*^ |
| **Difference in physical activity (min)** | 0.00  (-280.0 – 280.0) | 0.00  (-280.0 – 280.0) | 0.00  (-280.0 – 280.0) | 0.00  (-280.0 – 280.0) | 0.00  (-280.0 – 280.0) | 0.00  (-280.0 – 280.0) | 0.00  (-280.0 – 280.0) | 0.00  (-280.0 – 280.0) |
| **Increased physical activity** | 21.3 (215) | 19.4 (70) | 21.3 (210) | 19.6 (75) | 18.2 (178) | 27.5 (107)^**^ | 20.1 (203) | 23.0 (82) |
| **Reduced physical activity** | 43.0 (433) | 44.4 (160) | 57.8 (569) | 53.8 (206) | 43.2 (423) | 43.7 (170) | 42.0 (425) | 47.1 (168) |
| **Use of medication** | 73.9 (602) | 26.1 (213) | 71.6 (395) | 28.4 (157) | 69.6 (384) | 30.4 (168) | 72.5 (1010) | 26.1 (357) |

^1^ Mann-whitney was used in univariate analyses in continuous variables ^2^ Qui-square was used in univariate analyses in category variable

**Table 3.** Eating habit factors associated with eating behavior (uncontrolled eating, emotional eating and cognitive restraint) and stress among Brazilians during pandemic period in univariate analyses

| **Variables** | **Eating behavior** | | | | | | **Perceived**  **Stress** | | |  |  |
| --- | --- | --- | --- | --- | --- | --- | --- | --- | --- | --- | --- |
|  | **Uncontrolled eating** | | **Emotional eating** | | **Cognitive restraint** | | |  | | |  |
|  | **< 48.1** | **≥48.1** | **<61.1** | **≥61.1** | **<61.1** | **≥61.1** | | **<28.0** | **≥28.0** | | |
| **Difference in number of meals** | 0.00  (-6.0 – 4.0) | 0.00^**^  (-5.0 – 5.0) | 0.00  (-5.0 – 5.0) | 0.00^*^  (-6.0 – 5.0) | 0.00  (-5.0 – 5.0) | 0.00  (-6.0 – 5.0) | | 0.00  (-6.0 – 5.0) | 0.00  (-3.0 – 4.0) | | |
| **Increased number of meals** | 19.6 (198) | 32.8 (118)^**^ | 19.4 (191) | 32.6 (125)^**^ | 24.1 (236) | 20.6 (80) | | 22.5 (227) | 24.9 (89) | | |
| **Reduced number of meals** | 27.4 (276) | 24.4 (88) | 26.4 (260) | 27.2 (104) | 26.1 (256) | 27.8 (108) | | 24.2 (245) | 33.3 (119)^*^ | | |
| **Increased food intake** | 51.6 (520) | 78.3 (282)^**^ | 50.4 (496) | 79.9 (306)^**^ | 60.0 (587) | 55.3 (215) | | 57.5 (581) | 61.9 (221) | | |
| **Reduced food intake** | 18.4 (185) | 10.0 (36) | 18.7 (184) | 9.7 (37) | 14.3 (140) | 20.8 (81)^*^ | | 15.3 (155) | 18.5 (66) | | |
| **Increased snacking** | 44.3 (447) | 71.4 (257)^**^ | 42.8 (422) | 73.6 (282)^**^ | 52.7 (516) | 48.3 (188) | | 49.1 (496) | 58.3 (208)^*^ | | |
| **Reduced snacking** | 8.9 (90) | 5.6 (20) | 8.8 (87) | 6.0 (23) | 6.3 (62) | 12.3 (48)^**^ | | 7.1 (72) | 10.6 (38)^*^ | | |
| **Increased using food delivery service** | 46.2 (466) | 61.1 (220)^**^ | 46.5 (458) | 59.5 (228)^**^ | 51.0 (499) | 48.1 (187) | | 47.6 (481) | 57.4 (205)^*^ | | |
| **Reduced using food delivery service** | 14.8 (149) | 10.0 (36) | 14.1 (139) | 12.0 (46) | 14.4 (141) | 11.3 (44) | | 12.8 (129) | 15.7 (56) | | |
| **Increased homemade meals** | 66.4 (669) | 70.0 (252) | 65.8 (648) | 71.3 (273) | 65.4 (640) | 72.2 (281)^*^ | | 67.2 (679) | 67.8 (242) | | |
| **Reduced homemade meals** | 5.4 (54) | 7.2 (26) | 5.5 (54) | 6.8 (26) | 6.3 (62) | 4.6 (18) | | 5.4 (55) | 7.0 (25) | | |
| **Difference in food frequency intake** | | | | | | | | | |  |  |
| Legume | 0.00  (-10.0 – 10.0) | 0.00  (-10.0 – 10.0) | 0.00  (-10.0 – 10.0) | 0.00  (-10.0 – 10.0) | 0.00  (-10.0 – 10.0) | 0.00  (-10.0 – 10.0) | | 0.00  (-10.0 – 10.0) | 0.00  (-10.0 – 10.0) | | |
| Cereal | 0.00  (-10.0 – 8.0) | 0.00  (-10.0 – 10.0)^*^ | 0.00  (-10.0 – 10.0) | 0.00  (-10.0 – 10.0) | 0.00  (-10.0 – 10.0) | 0.00  (-10.0 – 8.0) | | 0.00  (-10.0 – 8.0) | 0.00  (-10.0 – 10.0) | | |
| Bakery products | 0.00  (-10.0 – 10.0) | 0.00  (-10.0 – 10.0) | 0.00  (-10.0 – 10.0) | 0.00  (-10.0 – 9.0) | 0.00  (-10.0 – 10.0) | 0.00  (-10.0 – 10.0) | | 0.00  (-10.0 – 10.0) | 0.00  (-10.0 – 9.0) | | |
| Milk and dairy | 0.00  (-10.0 – 8.0) | 0.00  (-10.0 – 10.0) | 0.00  (-10.0 – 10.0) | 0.00  (-8.0 – 10.0) | 0.00  (-10.0 – 10.0) | 0.00  (-10.0 – 8.0) | | 0.00  (-10.0 – 10.0) | 0.00  (-10.0 – 10.0) | | |
| Fruit | 0.00  (-10.0 – 10.0) | 0.00  (-9.0 – 10.0) | 0.00  (-10.0 – 10.0) | 0.00  (-9.0 – 10.0) | 0.00  (-10.0 – 10.0) | 0.00  (-9.0 – 10.0) | | 0.00  (-9.0 – 10.0) | 0.00  (-10.0 – 10.0) | | |
| Meat | 0.00  (-10.0 – 7.0) | 0.00  (-10.0 – 8.0) | 0.00  (-10.0 – 7.0) | 0.00  (-8.0 – 8.0) | 0.00  (-10.0 – 8.0) | 0.00  (-8.0 – 7.0) | | 0.00  (-10.0 – 7.0) | 0.00  (-10.0 – 8.0) | | |
| Canned products | 0.00  (-10.0 -10.0) | 0.00  (-10.0-10.0)^*^ | 0.00  (-10.0 -10.0) | 0.00  (-10.0 – 10.0) | 0.00  (-10.0 – 10.0) | 0.00  (-10.0 – 10.0) | | 0.00  (-10.0 – 10.0) | 0.00  (-10.0 – 10.0) | | |
| Vegetables | 0.00  (-10.0 – 10.0)^*^ | 0.00  (-8.0 – 10.0) | 0.00  (-10.0 – 10.0) | 0.00  (-10.0 – 9.0) | 0.00  (-10.0 – 9.0) | 0.00  (-10.0 – 10.0) | | 0.00  (-10.0 – 10.0) | 0.00  (-10.0 – 10.0) | | |
| Sugary drinks | 0.00  (-10.0 – 10.0) | 0.00  (-8.0 – 10.0)^*^ | 0.00  (-10.0 – 10.0) | 0.00  (-10.0 – 10.0)^*^ | 0.00  (-10.0 – 10.0) | 0.00  (-10.0 – 10.0) | | 0.00  (-10.0 – 10.0) | 0.00  (-10.0 – 10.0) | | |
| Instant meals and snacks | 0.00  (-10.0 – 10.0) | 0.00  (-10.0 – 10.0)^**^ | 0.00  (-10.0 – 10.0) | 0.00  (-10.0 – 10.0)^*^ | 0.00  (-10.0 – 10.0) | 0.00  (-10.0 – 8.0)^*^ | | 0.00  (-10.0 – 10.0) | 0.00  (-10.0 – 10.0) | | |
| Candies | 0.00  (-10.0 - 10.0)^**^ | 0.00  (-10.0 – 9.0) | 0.00  (-10.0 – 10.0) | 0.00  (-10.0 – 10.0)^**^ | 0.00  (-10.0 – 10.0) | 0.00  (-10.0 – 10.0)^*^ | | 0.0  (-10.0-10.0) | 0.00  (-10.0-10.0)^*^ | | |
| Fast food | 0.00  (-10.0 – 6.0) | 0.00  (-10.0 – 9.0)^*^ | 0.00  (-10.0 – 8.0) | 0.00  (-10.0 – 9.0)^*^ | 0.00  (-10.0 – 9.0) | 0.00  (-10.0 – 6.0) | | 0.00  (-10.0 – 9.0) | 0.00  (-10.0 – 6.0) | | |

^1^ Mann-whitney was used in univariate analyses in continuous variables ^2^ Qui-square was used in univariate analyses in category variables.
